# Thymus Regeneration and Future Challenges

**DOI:** 10.1007/s12015-020-09955-y

**Published:** 2020-01-29

**Authors:** Valentin P. Shichkin, Mariastefania Antica

**Affiliations:** 1 Bienta, Ltd, Chervonotkatska street 78, Kyiv, 02094 Ukraine; 2 Rudjer Boskovic Institute, 10000 Zagreb, Croatia

**Keywords:** Stem cells, Thymus, Thymectomised patients, Thymus regeneration, Thymic epithelial stem cells, Small chemical compounds

## Abstract

Thymus regenerative therapy implementation is severely obstructed by the limited number and expansion capacity in vitro of tissue-specific thymic epithelial stem cells (TESC). Current solutions are mostly based on growth factors that can drive differentiation of pluripotent stem cells toward tissue-specific TESC. Target-specific small chemical compounds represent an alternative solution that could induce and support the clonal expansion of TESC and reversibly block their differentiation into mature cells. These compounds could be used both in the composition of culture media designed for TESC expansion in vitro, and in drugs development for thymic regeneration in vivo. It should allow reaching the ultimate objective - autologous thymic tissue regeneration in paediatric patients who had their thymus removed in the course of cardiac surgery.

## Thymus Importance

Thymus is an essential organ of the immune system since it is the main site of T lymphocyte production and the place of adaptive immunity regulation. The thymus significance for development and function of the immune system is the centre of hot discussions since the 1961, when thymus function was first discovered by Jacques Miller [1]. Nowadays it is known that impaired thymus function may have a number of consequences for the immune system as an increased predisposition to infection and autoimmunity, reduced response to vaccines with age and possible risk of cancer development. Patients subjected to complete thymectomy as neonates are more likely to suffer from atherosclerosis, autoimmune or neurodegenerative diseases, as well as they have a higher predisposition to develop rashes, eczema, or contact allergies and show stable disbalance of naïve T cells in the periphery, especially if thymectomy happened at the age below one year [2–7]. According to other data, thymectomy has no critical clinical effects, if performed in the post-infant period [8]. However, most data were collected in a short follow up time after thymectomy and therefore not considering the time for the onset of age-related diseases in the thymectomised group. Also, the inclusion of individuals with residual thymic tissue might cause an underestimation of the impact of thymectomy.

Current epidemiological data indicate that almost 1 in 100 children is born with a congenital heart defect [6], and they are potential patients for heart surgery and partial or total thymectomy. Since thymectomy is a part of standard surgical procedure for congenital heart diseases, thymus becomes a medical waste, and in these cases, it may serve as an essential alternative source of autologous tissue-specific stem cells for personalized treatment of thymectomised infants, who are a high-risk cohort for many age-related diseases. In this relation, the collection and long-term storage of primary infant thymic tissue, as well as, the preparation and expansion of thymic epithelial stem cells (TESC) are very important issues that are discussed in this paper.

## Thymus Cell Architecture and Thymic Epithelial Cells

**Fig. 1.**
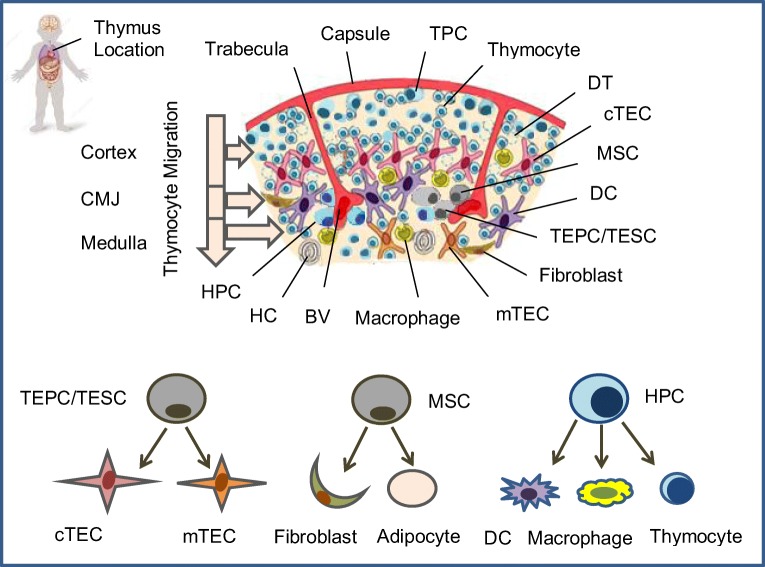
**Human thymus cell architecture.** The human thymus is located in the upper anterior part of the chest behind the sternum between lungs and lies on top of the heart along the trachea. The thymus reaches its maximum weight (about 28 gram) during puberty. This pinkish-gray organ consists of two lobes parted into lobules by connective tissue strands (trabeculae). Each thymic lobule has a cortex and medulla. Hematopoietic precursor cells (HPC) enters the thymus through postcapillary venules located at the corticomedullary junction (CMJ) and migrate to the capsule, committed CD4-CD8- T precursor cells (TPC) located in the subcapsular region, and immature CD4+CD8+ cortical thymocytes migrate through the cortex and CMJ to the medullar zone. The medulla contains CD4+ and CD8+ naïve thymocytes that will migrate to the periphery. The stromal-epithelial compartment of the thymus is represented by minor populations of EpCam+(CD326+)Foxn1+ bipotent thymic epithelial precursor cells/thymic epithelial stem cells (TEPC/TESC) and mesenchymal stem cells (MSC) located probably in the thymic parenchyma close to the CMJ region, as well as EpCam+CD205+ cortical thymic epithelial cells (cTEC) located in the cortex and EpCam+Air+ medullary thymic epithelial cells (mTEC) located in the medulla. Moreover, the cortex and the medulla contain also macrophages, fibroblasts and dendritic cells (DC) that together with cTEC and mTEC participate in the differentiation, maturation, positive and negative selection of thymocytes. HPC generate all thymocyte populations and alternatively may generate macrophages and DC; TEPC/TESC generate cTEC and mTEC lineages depending on local microenvironment and cross-talk with cortical or medullary thymocytes; MSC generate thymic fibroblasts and adipocytes. BV: Blood vessel; DT: Dead thymocyte; HC: Hassall’s corpuscle.

The thymus has a highly complex structure comprised of the thymic stroma and developing thymocytes (Fig. 1). The thymic stroma contains dendritic cells, macrophages, epithelial, mesenchymal and vascular elements [9–14]. In this multicellular structure with different cell types and functions several minor stem cell populations can be found, in particular thymic epithelial progenitor cells/thymic epithelial stem cells (TEPC/TESC) [15, 16•], mesenchymal stem cells (MSC) [17, 18] and lymphoid progenitor cells (LPC) [12, 19–24]. Of these, thymic epithelial cells (TEC) provide most of the specialist functions of the organ [25, 26]. As the thymus is organized into two regions, the cortex and the medulla, also TEC are defined according to their localization as cortical (c) and medullar (m) TEC. They are morphologically and functionally distinct, and they mediate different aspects of T cell development. The cTEC are required for commitment of early thymocyte precursors to the T cell lineage through provision of the Notch ligand Dll4 [13, 27] and to drive expansion of thymocytes at several stages of development through delivery of growth factors and cytokines [10, 25, 28••]. They also regulate positive selection of T cells through a unique set of peptides generated by a thymus-specific proteasome subunit, β5t [29]. The mTEC regulate migration of positively selected thymocytes from the cortex into the medulla, via expression of chemokines CCL19 and CCL21, and they also regulate the accumulation and positioning of dendritic cells in the medulla via secretion of the chemokine XCL1 [30]. Both pathways are also regulated by thymus resident dendritic cells, which are critical hematopoietic components of the thymus microenvironment [9, 12, 31]. An important role for the thymic tissue maintenance, differentiation and regeneration plays also the intrathymic radio-resistant LPC [19, 32–35], which probably relates to the stem cell population. Thus, production of a functional, self-tolerant T cell repertoire requires interactions between developing thymocytes and a variety of cortical and medullar TEC types derived from TEPC/TESC. Analysis of thymus development has established that cTEC and mTEC can originate from a common TEPC type in both, the fetal and postnatal mouse thymus, and their transplantation is sufficient to the functional establishment of the entire thymus [16, 36, 37]. In mice, these TEPC/TESC comprise 1–2% of total TEC and are located in the thymic parenchyma at the cortico-medullary junction. In mice they express Plet1, Ly51, and EpCAM (CD326) surface proteins [38••]. CD326 is also expressed in the fetal human thymus, and therefore, in combination with *Foxn1* expression could be used to identify the human TEPC/TESC population(s) [26, 39]. Recently, in the course of the ThymiStem project funded by European Union, Prof. Antica’s research group has detected epithelial precursors also from human thymus by using the stem cell ability to form spheres when cultured in non-adherent conditions in vitro (manuscript in preparation). This approach may become an alternative for the expansion of human functional *Foxn1*^+^ EpCAM^+^ TESC in vitro. In the mouse model thymospheres described by Ucar et al. were defined as formed from *Foxn1*^*−*^ thymic precursors [15]. However, according to more recent data, the thymospheres are formed by *Foxn1*^*−*^ EpCAM^−^ mesenchymal cells with the potential to generate only adipocytes, but no epithelial cells [40••].These mesenchymal cells might be important to the maintenance of the thymic microenvironment since it is already known that mesenchymal fibroblasts deliver growth factors to the developing TEC and cytokines to lymphocyte precursors. Therefore, thymospheres might be a stem cell population that maintains the non-epithelial microenvironment in the thymus. Since the data described are of mouse origin it is important to investigate more carefully also the human thymus model in vitro and in humanized mice.

## Thymus Reconstitution Strategies

The perspective for development of an effective thymus regenerative strategy is supported by the successful research on transplantation of in vitro cultured autologous thymic gland residues to DiGeorge syndrome patients [41, 42], generation of functional thymic epithelium from human embryonic stem cells (ESC) supporting host T cell development [43, 44], transplantation of mouse FOXN1-induced TEC [45], transplantation of mouse thymic pluripotent stem cells (PSC) [16], reconstitution of functional thymus organ culture in vitro [46] and transplantation of in vitro generated human artificial thymic organoids to humanized immunocompromised mice [47•]. Thus, current strategies for enhancing/restoring of the thymic function in patients arise mainly from studies on mouse experimental models and are based on i) enhancing the endogenous thymus regeneration [48]; ii) transplantation of thymic tissue [42]; iii) transplantation of pluripotent TESC/TEPC that generate thymic microenvironment in vivo or even may fully restore functional thymi [16, 45, 49]; iv) transplantation of thymic organoids grown in vitro that partially recapitulate thymus function [46] and v) transplantation of an artificial thymus created on a synthetic matrix [47•].

**Fig. 2.**
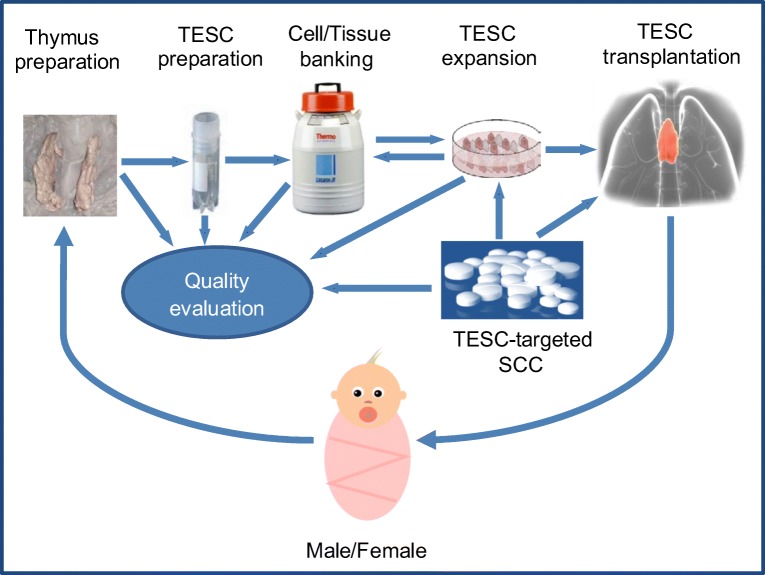
TESC/SCC-based strategy for thymus regenerative therapy in partially thymectomized infants. Thymic epithelial stem cell/Small chemical compound (TESC/SCC)-based strategy for autologous thymus regenerative therapy in infants could include the development of clinical grade protocols for collection, preparation and cryopreservation of primary infant thymic tissue and TESCenriched samples. These TESC could be used further to screen SCC for regulation, differentiation and proliferation of human TESC. The selected compounds would be tested for clonal expansion of TESC *in vitro* and for the reconstitution of thymic function *in vivo* in terms of maturation, differentiation and tolerance of autologous T cells as well as for supporting thymus tissue growth. Finally, full pharmacological evaluation of the properly selected and optimised compounds would be performed for high efficacy and low toxicity and further drug development. An actual challenge is the optimization of thymectomy procedure in infants to preserve a thymic fragment for consequent postsurgical thymus regenerative therapy. An additional impact on the efficacy of the post-surgical rehabilitation may provide the quality life monitoring of thymectomized patients in relation to their resistance to infections, allergies, autoimmune, oncological and other diseases associated with the impaired thymic function.

Thymus bioengineering is still at its early stage of development and more studies focusing on clinical-grade experimental conditions are needed to further advance the technology for medical applications. However, preclinical studies on mouse models have clearly proven that this is an effective approach for restoring and rejuvenating the function of the adaptive immune system by achieving the immunosuppression-free tissue/organ replacement [46, 47•]. Some preclinical and clinical studies aimed at the recovery of thymus function in vivo with the help of a variety of hormonal or cytokine treatments are already in progress [34]. Moreover several of these approaches have been tested in phase I or phase I/II clinical trials [25, 48–50]. In general, while current data suggest that some improvement in T cell numbers may result from these hormonal or cytokine-based therapies, major obstacles are high toxicity, low effectiveness and specificity, or significant negative side effects, and therefore currently a stable and effective reconstitution of the human thymus function is still elusive. Although, thymus transplantation studies demonstrated the utility of this procedure for restoring thymus function in patients, successful transplantations have only been established by using neonatal human thymus as autologous donor tissue [41, 42]. One study has shown that a microenvironment capable of supporting the early stages of T cell development can be generated by the introduction of four genes (Dll4, CCL25, KitL, and CXCL12) into Foxn1^−/−^ mouse thymic primordium [51]. This suggests the possibility of engineering a synthetic thymus based on the delivery of key molecules required for TEC to support T cell development in an artificial scaffold. Though, in our opinion, TESC-based cell transplantation approaches might be more appropriate for near-medium clinical goals, at least for the partially thymectomised infants (Fig. 2).

## Tissue-Specific TESC Versus iPSC and ESC

The potential of tissue-specific stem cells for treating incurable diseases and conditions is widely recognized through their capacity to restore tissue function by either cell transplantation or regenerative therapies. Stem cells underpin a number of modern therapies; however, all rely on transplantation of cells harvested ex vivo. The limited capacity to achieve a robust expansion of tissue-specific stem cells in vitro is recognized as a basic limitation for the development of new stem cell-based therapies. Furthermore, some human tissues, including the thymus, are not amenable to harvesting stem cells for autologous therapy either on grounds of tissue accessibility or the number of stem cells. Cell number in the thymus for instance may be limited by the size of the organ, or by age-related factors resulting in diminished cell numbers in adult and elderly patients. Strategies for clinical use of human TESC depend on the ability to generate or propagate undifferentiated TESC in vitro, and to control their differentiation in order to produce transplantable functional organoids or to support thymus regeneration in vivo for a complete recapitulation of sustained thymus function. They further require strong medical-grade procedures for thymic epithelial cell lines and cultures derivation, including protocols for cryopreservation of cultured cells and ex vivo tissue. Finally, they depend on the capacity to translate these issues from mouse models to human.

In spite of the current wide interest in human iPSC and ESC, and successful attempts to drive their differentiation in vitro towards mature tissue-specific cells [16, 43, 44], the conditions that are created in vitro are not fully equivalent to the in vivo conditions that are critically important for the final tissue-specific differentiation of such iPSC or ESC. Moreover, the use of ESC, as well as, their iPSC analogous cannot solve the transplantation challenges properly because of high risks of tumorigenicity and graft rejection, as well as regulatory, ethical and legal restrictions in most developed countries for the use of ESC in human transplantation/regenerative medicine. Taking into consideration these obstacles, postnatal tissue-specific stem cells, in particular, TESC are the preferable source for therapeutic purposes. Development of new approaches for their clonal expansion is an extremely relevant and important challenge that should be resolved in the nearest future.

## Expansion of Stem Cells In Vitro

One of the key challenges for stem cell biology is to develop the conditions that permit the expansion of functionally validated stem cells in vitro via self-renewal. Several strategies have been used to propagate defined tissue-specific stem cell types, but successful long-term cultures have been produced only for a very few tissue types, in particular, epithelial stem cells derived from skin and a variety of other organs are most effectively maintained as mixed cultures containing both stem cells and their differentiated progeny, using the specific murine feeder cell line 3 T3/J and keratinocyte stem cell conditions [52, 53]*.* The same protocol has been used to grow epithelial stem cells from a variety of tissues including the limbus/cornea [53, 54]. Intestinal epithelial stem cells can be maintained in long-term culture as organoids that contain both, the stem cells and their differentiated progenies [55]. In contrast, neural stem cells can be maintained under a variety of conditions as a near homogeneous stem cell population in a completely defined culture medium [56]. Recently, it was also described a chemically defined and growth-factor-free culture medium for the expansion and production of human PSC that contains just three small chemical compounds (SCC) with a much lower number of recombinant proteins than used in commercially available media [57•]. The long-term cell growth of non-transformed cell culture from adult mouse thymus was supported in vitro for about two years in the regular culture conditions in the presence of an autocrine thymocyte growth factor (THGF). These cells showed the properties of pluripotency, self-renewal, and tendency to form thymic organoids (thymospheres) in vitro, and were highly resistant to cortisol and gamma-irradiation [19, 32]*.* In vivo stem cells are maintained by a specific cellular microenvironment called the stem cell niche [58, 59]. Current understanding suggests that the self-renewal property of stem cells in vivo is determined by the proximity of the niche [60]. The goal of supporting the proliferation of self-renewing stem cells in vitro in long-term cultures essentially requires recreation of this niche in vitro, such that stem cells receive appropriate signals for proliferation in the absence of differentiation. Accumulated knowledge from many years of investigation led to the creation of completely defined conditions for growing mouse ESC [61] and human PSC [57•]. The basis of this protocol is that the major intracellular signaling pathway that normally promotes differentiation of PSC is blocked using a chemical inhibitor of MEK (mitogen-activated protein kinase) signaling while proliferation is maintained via the action of a glycogen synthase kinase (GSK) inhibitor. Moreover, the cells are maintained in a minimal essential medium containing N2B27, insulin, and transferrin [62]. These conditions preserve PSC in the pluripotent state and can also be used to grow iPSC. This approach, which combines blocking differentiation while promoting proliferation, should in principle be applicable to any stem cell population once the relevant signaling pathways are defined.

## Thymic Epithelial Cell Lines

For many years various laboratories have tried to grow functional TEC lines from primary mature TEC [63, 64]. However, such lines typically lose their functional capacity after only a short-term culture, rendering them useless for clinical aims. Furthermore, it is now clear that two types of mature TEC subpopulations (mTEC and cTEC) are required to fully support T cell development, and therefore growth of a single mature TEC sub-type will not be sufficient to develop thymus function [10, 25, 65]. Collectively, this indicates that TEC-based approaches must involve undifferentiated TEPC/TESC capable to produce all TEC subtypes of the mature organ. This establishes the rationale for developing protocols permitting in vitro expansion of functionally validated undifferentiated human TESC. Such cell lines would provide the optimum basis for thymic organoids, in which controlled differentiation of TESC results in production of all specific TEC populations required for full thymus regeneration. They could then be transplanted into patients to enhance the thymus function in vivo. However, the occurrence of a small number of TESC in human thymus, difficulties in their isolation, purification and especially expansion in vitro in undifferentiated and functional state as well as the preferential growth of fibroblasts in long-term cultures in vitro, still represent the major challenges for the study and possible application of the recovered tissue-specific stem cells [32, 47, 65, 66]. These problems yet remain unresolved. Current approaches that are exploring how to reach a substantial TESC growth in vitro include the use of serum-free culture media with TESC- supporting growth factors and other supplements which can inhibit the growth of other cell types [58, 67] as well as the use of low/non-adhesive materials and matrixes for 3D cultures [47, 60]. Human long-term TEPC/TESC cultures could be achieved using the 3 T3/J feeder-based keratinocyte stem cell conditions that were applied for stem cell cultures from many types of epithelial tissues including thymic tissue [52, 53]. While cells grown under these conditions can contribute to epithelial networks*,* they do so at low efficiency and further optimization for an increase of the functional stem cell frequency is required to develop clinically useful lines. Functional cultures of thymic stromal-epithelial stem cells can be derived also in low-adhesive conditions as cultures of thymospheres [15, 40] or in adhesive conditions as cultures of thymic explants and monolayer cultures [39, 65, 66]. These thymus-derived cell cultures contain both TESC and their differentiated progenies as well as MSC and fibroblasts. Importantly, some of the cultured cells also retain the capacity to contribute to thymic stromal-epithelial networks where they exhibit a normal thymic function in terms of T cell differentiation from CD34+ hematopoietic stem cells [39, 66] and Antica, unpublished data. Thus, the current culture conditions, which are optimized for epithelial stem cells can be used as a starting point to define optimal conditions for an effective support of the human TESC growth. The current goal is to establish fully defined feeder-free culture conditions, in particular by using chemical compounds as signaling pathway inhibitors.

## **TESC-Specific Small Chemical Compounds** (SCC)

A promising approach is the use of SCC that could block or enhance the signalling mediated by specific protein-kinases and thus regulate differentiation and clonal expansion of stem cells or even reprogram fibroblasts into ESC [57•, 68, 69••]. A number of such target-specific compounds were already screened and tested using high throughput screening (HTS) assays with human ESC or iPSC, as well as HSC and MSC isolated from bone marrow or cord blood [69••, 70••, 71••]. These studies provided highly promising results validating the use of SCC in regenerative medicine both, for tissue engineering in vitro and for boosting regenerative potential of stem cells in vivo*.* However, optimal compounds for TESC have yet to be identified and structurally optimized to achieve adequate efficiency and low toxicity in vitro and in vivo, and other benefits for the patients and the industry. Finding and optimization of new effective compounds will allow to expand primary isolated single human TESC or even to reverse mature mTEC or/and cTEC to their common stem cell precursor. Therefore, this goal is highly attractive, because it provides new high relevant approaches for further compound-based development of TESC-specific drugs that should be non-toxic in vivo, inexpensive and convenient be used by the patients. These advantages are in contrast with traditional biological tool applications, such as growth factors, which are expensive, easily degradable, and have a number of side effects in vivo at their therapeutically relevant concentrations. Furthermore, these compounds can be highly attractive as supplements to culture media specifically designed for TESC attempted for clinical use. Finding new TESC-specific compounds will allow the replacement of expensive and unstable growth factors in culture media or at least the reduction of their concentrations. A similar compound-based cytokine-free culture medium composition has already been described for human PSC [57•]. Once the TESC-specific compounds are developed and primary TESC expanded, they could be extensively used for further cryopreservation studies, development of thymic organoids and pre-clinical transplantation studies.

The general strategy for the discovery of new target-specific SCC candidates for drug development is well established [72–75]. It is a long and complicated multi-stage process that requires large efforts and capital investments. In recent years the use of HTS technologies for large and structurally diverse chemical libraries led to the fast progress in the identification of lead compounds with therapeutic activities against a multitude of molecular targets and pathways [76, 77]. However, while several key target molecules that are critically important for human thymus development and function have been described, we are not aware of any specific research involving human TESC/thymic tissue models for screening TESC-specific compounds. We believe that promising molecular stem cell targets for such HTS assays are the retinoblastoma (Rb) protein family (pRb1/105, p107, and pRb2/p130). It is known that a homeostatic level of Rb activity is essential for self-renewal and survival of human embryonic stem cells (ESC) [78]. Rb inactivation prevents thymus involution and promotes thymic function by a direct control of FOXN1 gene expression [79]. FOXN1 is dynamically regulated in TEC during embryogenesis and at the onset of thymic involution; in particular it is highly expressed in TESC and is not expressed in non-functional TEC [26]. Thus, FOXN1 plays a critical role in thymus development, function, maintenance, and regeneration, which characterises it as a master regulator of TEC differentiation [80]. Efficient commitment of human ESC to the thymic epithelial precursor lineage can be achieved by precisely regulating the activities of tumour growth factor β (TGFβ), BMP4, retinoic acid (RA), Wnt, Sonic Hedgehog (Shh), and FGF signalling throughout differentiation [43, 81]. Thus, at least some of these targets can be used for the identification of new compounds that may efficiently regulate the proliferation and differentiation of human TESC in vitro and/or stimulate the regeneration of human thymus in vivo.

While the vast majority of compound screens related to human stem cells were performed using ESC and iPSC or HSC, mainly due to the restricted access to tissue-specific stem cells and a significant challenge in expanding them in vitro, a well-established access both, to the paediatric thymic tissue as a tissue-specific source of human TESC, and access to the vast and diverse SCC libraries will define the further progress in the discovery of TESC-specific compounds and development of drugs for thymus regenerative therapy (Fig. 2).

## Thymic Tissue Cryopreservation

The aim of effective TESC cryopreservation is in line with common cryopreservation problems for human stem cells derived from different sources, and it is in the process of consequent solving by research groups both, from academy and industry. While bone marrow and cord blood are the primary sources of HSC and MSC, and protocols for cryopreservation of these cell types are well established [82–85], there are only few reports for the human thymic tissue cryopreservation [65, 86, 87]. Currently, various freezing media designed specifically for cryopreservation of stem cells are available from different manufacturers, and some of these were applied for the cryopreservation and the long-term storage of the human thymic tissue in liquid nitrogen [65, 87]. A specific investigation concerning cryopreservation of postnatal thymic tissue in a wide range of cryoprotective conditions has been implemented by Prof. Shichkin’s research group during the ThymiStem project [39, 65]. In this study, the influence of a number of cryoprotective media with either penetrating (DMSO, glycerol) or non-penetrating (dextran-40, sucrose, hydroxyethyl starch) components was evaluated, and compared to the commercial GMP manufactured cryoprotective medium Stem-CellBanker (AMS Biotechnology, UK). Stem-CellBanker is serum-free and DMSO-containing medium, and it was created specifically for stem cell storage. This study indicated that for either cell suspensions or thymic fragments, the best combination for long-term storage was DMSO and dextran-40 (CPM-7) as judged by the CD326^+^ epithelial cells’ viability and formation of a stromal-epithelial cell monolayer after thawing [39, 65]. This cryoprotective medium could be further optimized specifically for human TESC in comparison with the set of available commercial media. Further, a favorable component for cryopreservation experiments involving human TESC is the Rho-associated protein kinase (ROCK) inhibitor, which greatly increases cell viability [88]. However, there is a great need for further optimization of cryopreservation protocols specifically for human TESC in accordance with the clinical requirements (Fig. 2).

## Gender Issues

Sex analysis in the context of human diseases and drugs discovery research has revealed clinically significant differences in pathophysiology between women and men. Female sex and age comprise two important risk factors for altered drug exposure and response [89]. Evaluation of the sex as a factor of a biological variable in basic biomedical and preclinical/clinical research is considered as an important methodological component of study design [90–93]. At this, researchers should consider both the sex of the patient/animal experimental groups for study in vivo and the sex of tissue/cells for study in vitro [91, 94]. Since these sex and gender differences exist, a special attention should be paid to thymic tissue collection from infants with congenital heart diseases and considering the appropriate balance between male and female representatives as part of the study to analyze sexual differences in TESC response to SCC action in vitro*.* Further, the influence of culture conditions on proliferation and differentiation of male and female human TESC should be considered. The gender and sex aspects will impact on the research design and a common strategy of the thymus-specific compound selection and further pharmacological studies in vivo with the use of small rodents. For testing the ADME/T (absorption, distribution, metabolism, excretion, and toxicity) properties the selected SCC in vitro with the use of tissue/cell models should be also taken into account to achieve the highest benefit and lowest risk for the patients (Fig. 2).

## Ethics and Framework Conditions

Cell therapy is one of the major prospects in current scientific and medical development. However, elements or products of the human body are normally considered in a number of countries as being protected from any form of commercialisation. Thus, there’s a number of possible problems involving human cells from donors, the nature and limits of adequate exploitation, obtaining informed consent on their use, and a possible conflict of interest between patients, stakeholders, scientists and society. In agreement with recommendations of Regulatory agencies such as the US Food and Drug Administration (FDA) [95] and also the European Medicines Agency (EMA) [96] donors of human tissue or cells ought to be tested and screened for infections, the informed consent of patients or their legal guardians should be received before tissue/cell donation, and the entire technological process ought to be achieved in compliance with good laboratory practice (GLP) or good manufacturing practice (GMP) [97].

In case of allogeneic use of the cells, the donor should give a written and legally valid informed consent that covers possible research and therapeutic findings and commercial application. It should be ensured that the patients or their legal guardians sufficiently comprehend the stem cell-specific aspects of their participation in the research. Donors should be screened for infectious diseases and other risk factors, as it is recommended for blood and solid organ donation, and also for genetic diseases if appropriate. GLP and regulatory guidelines related to human tissues and cells should always be followed (Fig. 2). In preclinical studies appropriate in vitro and/or animal models ought to provide evidence of product safety in agreement with the Declaration of Helsinki and the Nuremberg Code. Also, in compliance with the Animal Welfare Recommendation, in vitro procedures should replace animals whenever possible. Since clinical research is indispensable for the final efficacy assessment of the cell-based treatment, it is important to protect human rights and welfare during this process and rigorous review pathways should make sure that stem cell-based products adapt to the best standards of evidence-based drugs, consistent with legal requirements for evidence-based medicine.

## Outputs from ThymiStem

Additionally to the discussion above, the main advances resulting from the ThymiStem project funded under FP7 Health for 2013–2017 (Project ID: 602587) lay the foundations necessary to recover thymus formation using stem cell-based bioengineering. ThymiStem was the European Consortium for “Development of Stem Cell-Based Therapy for Thymic Regeneration” comprised by 8 research teams from 6 countries (Great Britain, Spain, Czech Republic, Croatia, Ukraine and USA) coordinated by Prof. Clare Blackburn (The University of Edinburgh, UK). The more detailed description is provided in the Final Report Summary [98].

## Future Perspectives

The primary mission of the subsequent research should be addressed to further develop and advance the methodology for regenerating thymic function in patients who were subjected to partial or total thymectomy. At these, the main focus should be on infants at age range up to 12 months for whom the thymus regenerative therapy is fully justified, and the prove-of-concept is provided. Suitable solutions for clonal expansion and long-term cryopreservation of human thymus and TESC as well as the detection of new TESC-specific compounds and a subsequent development of thymus-specific drugs based on these compounds are extremely important steps to reach the final objective - immunorehabilitation of thymectomised patients in the course of the postsurgical therapy. This aim is fully realistic and achievable within the near future (Fig. 2). Moreover, thymus regenerative technology can be expanded further for a larger group of patients, including elderly people with age-related thymic involution and decreased thymic function, or chemotherapy-treated patients and, in some cases, for patients with removed thymoma and thymus-associated autoimmune diseases. Thus, delivering this technology to the end-users can significantly reduce medical costs and improve postsurgical rehabilitation therapy of recently thymectomised infants as well as improve their life quality in a long-term. Furthermore, this technology may provide sufficient impact on the life quality in elderly populations with deficiency of thymic functions and finally stimulate the creation of first thymus biobanks to provide support for personalised autologous thymus regenerative therapy.

## Executive Summary

### Thymus Importance

- Patients undergoing complete thymectomy at the age below one year may have a number of pathological consequences for the immune system, and they are more likely to suffer from age-related diseases.
- The thymus may serve as a source of autologous tissue-specific stem cells for thymectomised infants.

### Thymus Cell Architecture and Thymic Epithelial Cells

- Cortical and medullary thymic epithelial cells can originate from a common thymic epithelial precursor/stem cells (TEPC/TESC), which are sufficient to the direct establishment of the entire thymus microenvironment for T cell development.

### Thymus Reconstitution Strategies

- Enhancing of the endogenous thymus regeneration or transplantation of thymic tissue, pluripotent TEPC/TESC, thymic organoids and artificial thymuses are current thymus reconstitution strategies.
- TEPC/TESC-based cell transplantation approaches are more appropriate for partially thymectomised infants.

### Tissue-Specific TESC Versus iPSC and ESC

- Autologous tissue-specific stem cells are a preferable source for stem-cell-based regenerative therapy.

### Expansion of Stem Cells In Vitro

- In vivo stem cell self-renewal is determined by proximity to the specific cellular microenvironment called the stem cell niche.
- Recreation of the stem cell niche in vitro is a perspective goal for supporting the proliferation of the self-renewing stem cells in culture.

### Thymic Epithelial Cell Lines

- The current goal is to establish fully defined feeder-free culture conditions for human TESC using chemical compounds as signalling pathway inhibitors.

### TESC-Specific Small Chemical Compounds

- Optimal TESC-specific compounds have yet to be identified and structurally optimized to achieve adequate efficiency and low toxicity for the expansion of human TESC in vitro and the thymic regeneration in vivo.

### Thymic Tissue Cryopreservation

- Protocols for cryopreservation and quality evaluation of human thymic tissue/TESC should be optimized in accordance with the clinical requirements.

### Gender Issues

- Evaluation of the sex as a factor of the biological variable is an important methodological component of the study design.
- Both sexes of patient/animal experimental groups for study in vivo and the sex of tissue/cells for study in vitro should be considered.

### Ethics and Framework Conditions

- A complex of ethical problems includes the nature and limits of acceptable commercialization of paediatric thymic tissue/TESC and a conflict of interest between patients, investors, donors, researchers, and society.

### Outputs from ThymiStem

- ThymiStem demonstrated sufficient progress toward thymus regenerative therapy on molecular, cellular and bioengineering levels.

### Future Perspectives

- Further methodology development for thymic function regeneration in thymectomised patients should be addressed in forthcoming research projects.
- Thymus regenerative therapy is fully justified for infants thymectomised during heart corrective surgery and it should be the main focus of future research.

## Financial & Competing interest’s Disclosure

This paper derives from the ThymiStem project that was funded by the European Union’s Seventh Programme for research, technological development and demonstration under the grant agreement No [602587] for 2013–2017, the Scientific Centre of Excellence for Reproductive and Regenerative Medicine (project “Reproductive and regenerative medicine - exploration of new platforms and potentials”, Grant Agreement KK01.1.1.01.0008 which is funded by the European Union through the European Regional Development Fund), Terry Fox Foundation and InnovaTRT project that was submitted for funding to the European Union’s Horizon 2020 Programme for 2019–2024 (Proposal number: 874614). The authors have no other relevant affiliations or financial involvement with any organization or entity with a financial interest in or financial conflict with the subject matter or materials discussed in the manuscript apart from those disclosed.
